# Introducing functional dysplasia: Dynamic pelvic mechanics during running reduce femoral head coverage

**DOI:** 10.1002/jeo2.70711

**Published:** 2026-04-14

**Authors:** Renato Locks, Eliane C. Guadagnin, Felipe F. Gonzalez, Guilherme Pradi Adam, Jorge Chahla, Andrew E. Jimenez, Robert F. LaPrade, Leonardo Metsavaht, Gustavo Leporace

**Affiliations:** ^1^ Escola Paulista de Medicina Universidade Federal de São Paulo São Paulo Brazil; ^2^ Instituto Brasil de Tecnologias da Saúde (IBTS) Rio de Janeiro Brazil; ^3^ Rush University Medical Center Chicago Illinois USA; ^4^ Clínica Imagem Florianópolis Brazil; ^5^ Yale School of Medicine New Haven Connecticut USA; ^6^ Twin Cities Orthopedics Edina Minnesota USA

**Keywords:** biomechanics, borderline dysplasia, femoroacetabular impingement, gait, pelvic mechanics

## Abstract

**Purpose:**

To investigate the influence of peak contralateral pelvic drop and ipsilateral femoral adduction during running on traditional radiographic parameters of femoral head coverage.

**Methods:**

This cross‐sectional retrospective study included 19 patients (38 hips), 9 males and 10 females, with a mean age of 40 ± 10 years, all presenting with symptomatic unilateral femoroacetabular impingement. Participants underwent a three‐dimensional running analysis and anteroposterior pelvic radiographs. Using specialised software, the femur and pelvis were rotated in the coronal plane based on the peak angles of contralateral pelvic drop and femoral adduction obtained from the biokinetic running analysis. After prior validation, traditional femoral head coverage parameters were assessed on both standard and adjusted radiographs, and the variation of each radiographic measurement was obtained.

**Results:**

The mean contralateral pelvic drop and ipsilateral femoral adduction were 4.6° ± 3.8° and 5.3° ± 2.6°, respectively. Comparing adjusted with standard radiographs, the lateral centre‐edge angle significantly decreased by 4.8° ± 4.1° (*p* < 0.001), while the femoro‐epiphyseal acetabular roof index, acetabular index, sharp angle and extrusion index significantly increased by 10.1° ± 5.9°, 5.4% ± 4.4%, 5.2° ± 4.5° and 4.7° ± 4.0° (*p* < 0.001), respectively. After image adjustment, the percentage of hips classified as dysplastic increased based on the lateral centre‐edge angle (0%–18%), acetabular index (11%–45%), extrusion index (5%–21%) and sharp angle (45%–76%). The femoro‐epiphyseal acetabular roof index classified five hips (13%) as unstable. Linear regression demonstrated that each degree of pelvic drop resulted in a decrease of 0.92° of the lateral centre‐edge angle and increases of 0.94°–1.13° in the other radiographic measurements.

**Conclusion:**

This study demonstrated that femoral head coverage significantly decreases due to contralateral pelvic drop during running. This results in an increased number of dysplastic findings on adjusted radiographs. Patients with significant contralateral pelvic drop and ipsilateral femoral adduction during running may be at risk for functional dysplasia.

**Level of Evidence:**

Level III, retrospective cross‐sectional.

**Abbreviations**
AI: acetabular index
AP: anterior‐posterior
CAST: Calibrated Anatomical Systems Technique
EI: extrusion index
FAD: femoral adduction
FAIS: Femoroacetabular impingement syndrome
FEAR index: femoro‐epiphyseal acetabular roof index
LCEA: lateral centre‐edge angle
OA: osteoarthritis
PD: pelvic drop
SA: sharp angle

## INTRODUCTION

Femoroacetabular impingement syndrome (FAIS), according to the Warwick agreement, is defined as a triad of symptoms, clinical signs of impingement and corresponding imaging findings [11]. It represents symptomatic premature contact between the acetabular rim and the proximal femur. The literature reports that FAIS and acetabular dysplasia are the two main causes of early hip degeneration [22]. Consequently, proper diagnosis and distinction between dysplasia and FAIS are critical in order to improve function and potentially prevent osteoarthritis (OA) progression [25].

Imaging plays a key role in the diagnosis and assessment of patients with FAIS and hip dysplasia. In this context, the conventional anterior‐posterior (AP) pelvic radiographic view represents the most validated diagnostic tool [33, 36]. A variety of morphometric measurements can be extracted from a simple AP pelvic radiograph, including the lateral centre‐edge angle (LCEA), acetabular index (AI), also known as Tonnis angle, sharp angle (SA), extrusion index (EI) and the femoro‐epiphyseal acetabular roof index (FEAR index). Reliable radiographic techniques and accurate measurements are fundamental when evaluating patients, assisting in the decision‐making of operative and nonoperative treatments, and optimising outcomes in hip preservation surgery [12, 24]. However, most previously validated measurements are performed on standard static radiographs, which do not account for the dynamic changes in the musculoskeletal system that occur during everyday movements.

In this context, biokinetic analysis (3D motion integrated with strength and range of motion) [26] has become a valuable tool in the assessment of musculoskeletal motion and the identification of factors associated with orthopaedic injuries [1, 7, 21]. A previous study reported that runners with orthopaedic injuries exhibited excessive contralateral pelvic drop (PD) and femoral adduction (FAD) when compared to healthy controls [4]. These findings underscore the potential of in vivo functional analysis to enhance diagnostic accuracy and allow for a comprehensive evaluation of patient symptoms. More recently, efforts have been made to integrate dynamic motion analysis with radiographic assessments to better understand the behaviour of acetabular coverage during movement. This integrated approach has shown promising results with validated methods demonstrating reliable measurement of key parameters related to femoral head bone coverage [23]. However, while three‐dimensional motion analysis enables precise characterisation of pelvic and femoral kinematics during activities such as running, there is currently no standardised or clinically validated classification of hip dysplasia based on 3D kinematic findings. In contrast, conventional radiographic parameters remain directly linked to diagnosis, treatment planning and clinical outcomes [14].

A previous study suggested that femoral coverage measurements during a standing position may approximate coverage during normal gait in healthy subjects [39]. However, there is a lack of studies evaluating how contralateral PD and FAD can influence the femoral head bone coverage during running. The purpose of this study was to conduct a cross‐sectional investigation on the influence of peak contralateral PD and FAD during running on the traditional AP pelvis radiographic parameters (LCEA, AI, SA, EI and FEAR index) in individuals presenting with unilateral FAIS. The hypothesis was that radiographic parameters related to femoral head coverage would demonstrate reduced coverage when adjusted for PD and FAD, compared with traditional static radiographs.

## METHODS

### Participants

This is a retrospective cross‐sectional study (level III of evidence) involving patients who had undergone biokinetic analysis and standardised supine AP pelvic radiographs. All patients with a prior diagnosis of symptomatic unilateral FAIS between 2020 and 2023 were eligible for inclusion in the study. FAIS diagnosis was defined based on the Warwick agreement by a single fellowship‐trained orthopaedic surgeon with more than 10 years of experience through the presence of a triad of symptoms, clinical signs and image findings [11]. This study was approved by the Universidade Federal de São Paulo Ethics Committee under the number 7.079.076.

Patients with previous hip or spine surgeries, signs of hip OA (Tonnis Grade >1) and dysplasia (LCEA < 20°), or previously documented lower limb length discrepancies greater than 1 cm were excluded to avoid any factor that could influence the pelvic and lower limb biomechanics. Further, patients with poor‐quality radiographs according to the European guidelines on quality criteria for diagnostic radiographic images were excluded [9].

### Biokinetic analysis

The biokinetic analysis was conducted following a methodology previously described in the literature [23]. The biokinetic analysis was performed during running utilising four high‐frequency Qualysis cameras (Göteborg, Sweden) with an acquisition frequency of 250 Hz. As previously described, the reflective markers were placed on anatomical landmarks [23]. After marker positioning, a static trial was conducted, according to the Calibrated Anatomical Systems Technique [6], utilising a pointer equipped with two markers for landmark registration, determining the calibration, anthropometric and inertial parameters in orthostatic posture for each patient. Lastly, a functional calibration was performed to calculate hip and knee joint centres [5, 8].

**Figure 1:**
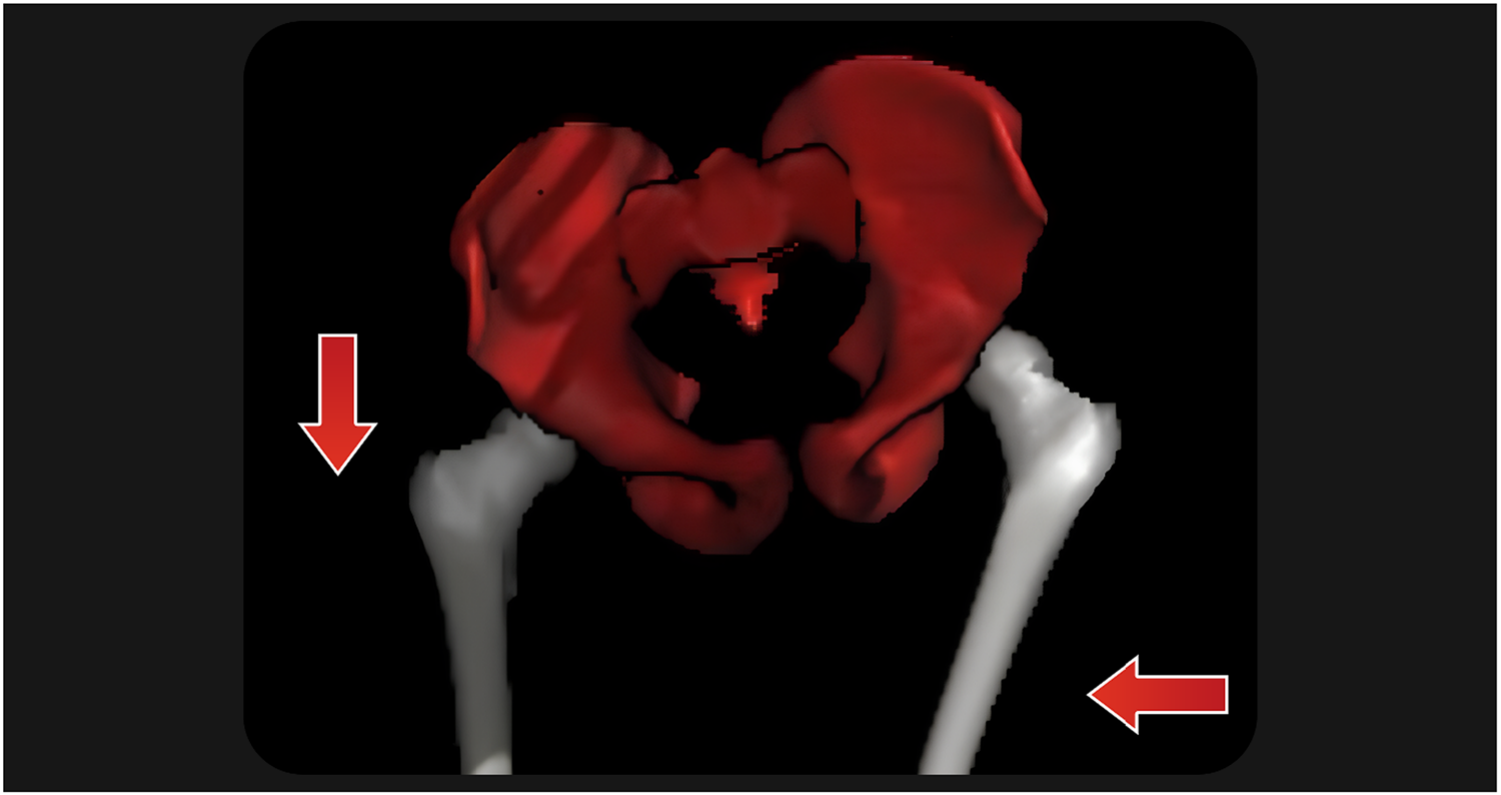
Three‐dimensional reconstruction generated from the biokinetic analysis depicting a left‐side femoral adduction and contralateral pelvic drop.

The participants ran with their regular running shoes and at a speed of their choice on a treadmill (ProForm Pro 2000, ProForm). The speed ranged from 8 to 12 km/h, according to patient preference. Two trials of 1 min were collected after a 3‐min warmup, totalling 5 min of testing, in accordance with previous protocols [28, 29]. The peak of contralateral PD for each side during the stance phase of running was determined and extracted. Similarly, the degree of FAD at the moment of peak PD was also recorded (Figure 1).

### Image acquisition and adjustment

Image acquisition and adjustment were performed using a standardised protocol, consistent with methods described in a previous study [23]. A standardised technique was used for supine AP pelvis radiographic acquisition. The film‐focus distance was 120 cm for both views. The centre of the x‐ray beam was directed to the midpoint of the symphysis and a line connecting the anterosuperior iliac spines [9, 37]. The AP pelvis radiograph was used to calculate the radiographic parameters. The images were numbered and shared with the investigators without any patient identification in Digital Imaging and Communications in Medicine format files.

The AP pelvis radiographs were uploaded, and adjusted using the Adobe Photoshop Software (Adobe Photoshop. Version 22.0.1, Adobe Systems Incorporated, 2012) under the supervision of a senior orthopaedic surgeon and an Adobe Photoshop expert.

**Figure 2:**
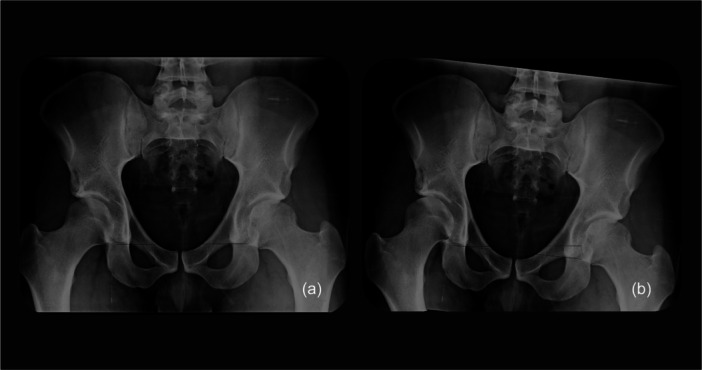
(a) Antero‐posterior (AP) pelvis radiograph. (b) Pelvis radiograph showing the adjusted radiograph with femur and the pelvis rotated according to the peak angles of pelvic drop and femoral adduction extracted from the coronal plane running biokinetic analysis (right hip).

Using Adobe Photoshop, a line connecting the bottom of both teardrop lines was drawn. Next, the femur was segmented from the radiographic image, keeping the native rotational centre of the hip joint. The peak angles of PD and FAD extracted from the running biomotion analysis were used to determine the degree of coronal rotation of the pelvis and femur, respectively. Following this, a new line was drawn at the same position of the native teardrop line. This horizontal line represented the ground surface, which becomes the reference during motion. The same procedures were repeated for the contralateral side (Figure 2a,b).

### Radiographic measurements

Carestream software (Carestream Software, Version 12.0, Carestream Health, 2023) was used to perform the five measurements described below on both hips, conducted by a senior musculoskeletal radiologist with more than 15 years of experience. This methodology of incorporating dynamic motion into femoral head acetabular coverage radiographic measurements has been recently validated, providing reliable assessments for most radiographic parameters [23].

**Figure 3:**
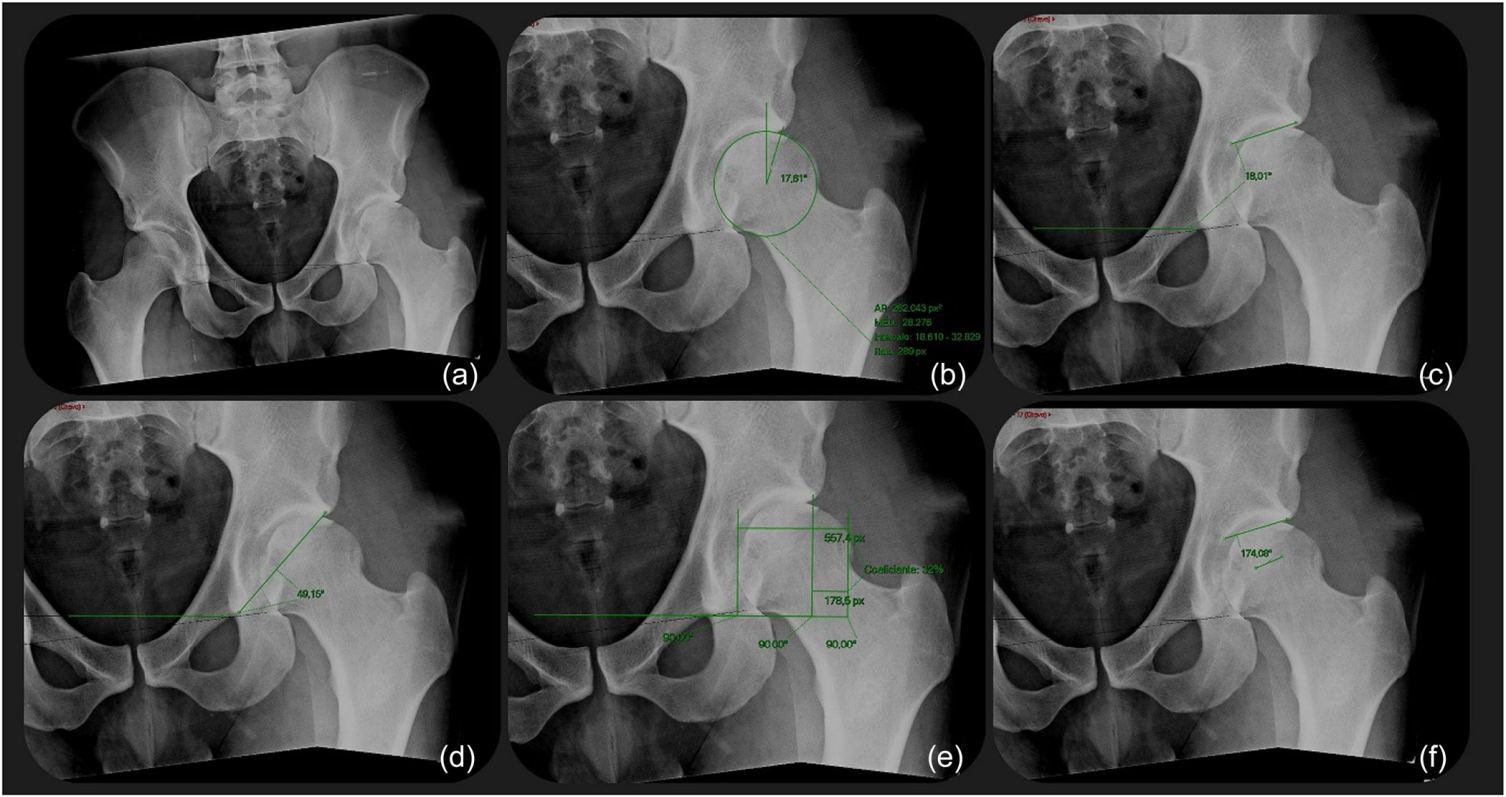
Adjusted radiographs. (a) Anteroposterior (AP) pelvis standard X‐ray. (b) Lateral centre‐edge angle (LCEA). (c) Acetabular index (AI). (d) Sharp angle (SA). (e) Extrusion index (EI). (f) Femoro‐epiphyseal acetabular roof (FEAR) index.

First, all measurements were performed on the standard AP pelvis radiograph for all hips and recorded in an Excel file called ‘standard radiograph’. Second, without looking at previous values, the same angles and measurements were performed on the adjusted radiographs for each hip (symptomatic and asymptomatic). These values were recorded in a different file named ‘adjusted radiographs’. The variation index for all five measurements was obtained by subtracting the standard by the adjusted radiographic values (Figure 3a–f).

All measurements on the standard radiographs followed the original definitions. The LCEA comprises the angle created by a line connecting the centre of the femoral head to the lateral edge of the acetabular sourcil, alongside a line that runs perpendicular to the longitudinal pelvic axis, also known as the teardrop line. The AI, or Tonnis angle, is defined as the angle formed between a line extending through the most medial point of the sclerotic zone of the acetabular roof and the lateral edge of the acetabulum, in relation to a horizontal line at the inferior margin of the pelvic teardrop. The SA is determined by the angle between a line from the teardrop to the lateral edge of the acetabulum and a horizontal line at the inferior margin of both teardrops. For the adjusted radiographs LCEA, AI and SA measurements, the initial supine teardrop line was used as a reference. The EI represents the percentage of the femoral head that is uncovered compared to its total diameter [36]. Lastly, the FEAR index is measured as the angle between a line through the most medial point of the sclerotic zone of the acetabular roof and the lateral edge of the acetabulum, intersecting with the central part of the physeal scar of the femoral head growth plate [42].

Patients were categorised as dysplastic based on various radiographic measurements on the standard radiographs. For the LCEA classification, hips with angles less than 20 degrees were considered dysplastic, those between 20 and 25 degrees considered borderline dysplastic, angles from 25 to 40 degrees classified as normal coverage and angles exceeding 40 degrees identified as over‐coverage [13, 16]. The AI has a normal range of −10 to 10 degrees, with angles greater than 10 degrees classified as dysplastic and those below −10 degrees categorised as pincer type [16]. For the SA, measurements below 32 degrees were deemed insignificant, while angles from 32 to 39 degrees were considered normal, those from 39 to 42 degrees classified as borderline, and angles exceeding 42 degrees identified as dysplastic [33]. The EI was regarded as normal when below 25% and dysplastic when at or above this threshold [34, 43]. Lastly, the FEAR index indicated hip stability, with values of 5 degrees or higher classified as unstable and those below 5 degrees considered stable [42]. The same classifications for each radiographic measurement were used for reclassification after image adjustment, due to the lack of dynamic radiographic classifications and also because these classifications are used routinely in decision‐making and surgical planning.

### Statistical analysis

The sample size was determined using the data comparing the standard and the adjusted images from a previously published study. This previous study analysed the intra and interrater reliability for the same measurements [23]. All the measurements were used to determine the sample size, and the largest value was utilised here, which were found for the LCEA (standard = 29.3 ± 7.0, adjusted = 25.1 ± 6.8, effect size = 1.14) and for the EI (standard = 0.15 ± 0.06, adjusted = 0.21 ± 0.07, effect size = 1.16), assessed each by one individual rater in the previous study (1st assessment of rater 1 and 2nd assessment of rater 2, respectively). Thus, nine hips were the minimal number of hips to achieve 80% statistical power with an alpha level of 0.05. The sample size was determined using the G*Power software (version 3.1).

Means, standard deviations and frequencies were utilised to describe the data. Data normality was determined using the Shapiro‐Wilk test. Both the symptomatic and asymptomatic limbs demonstrated no significant differences in PD or FAD (*p* = 0.847 and *p* = 0.536, respectively). Therefore, both symptomatic and asymptomatic hip measurements were included in the statistical analyses. Thus, to compare the standard versus the adjusted images, paired *t*‐tests or Wilcoxon tests were performed. The correlation between the variation of the image parameters and the PD and FAD during running was tested using the Pearson test. The correlations were classified as follows: *<*0.1: trivial; 0.1–0.3 small; 0.3–0.5 moderate; 0.5–0.7 large; 0.7–0.9 very large; >0.9 nearly perfect [15].

The proportion of dysplastic patients for the standard and the adjusted images were compared using the chi‐squared test. Simple and multiple linear regressions were utilised to identify if the PD and FAD could explain the variation in the adjusted images. Only those parameters with statistically significant correlation were utilised in the regression models. We tested the correlation of PD and FAD with running speed and BMI. As the results showed no significant correlations, we did not include them as covariates in the models. For all statistical tests, a significance level of 5% was considered. The software Jamovi (The Jamovi project, 2023, Jamovi Version 2.3) was utilised for statistical analysis.

## RESULTS

**Figure 4:**
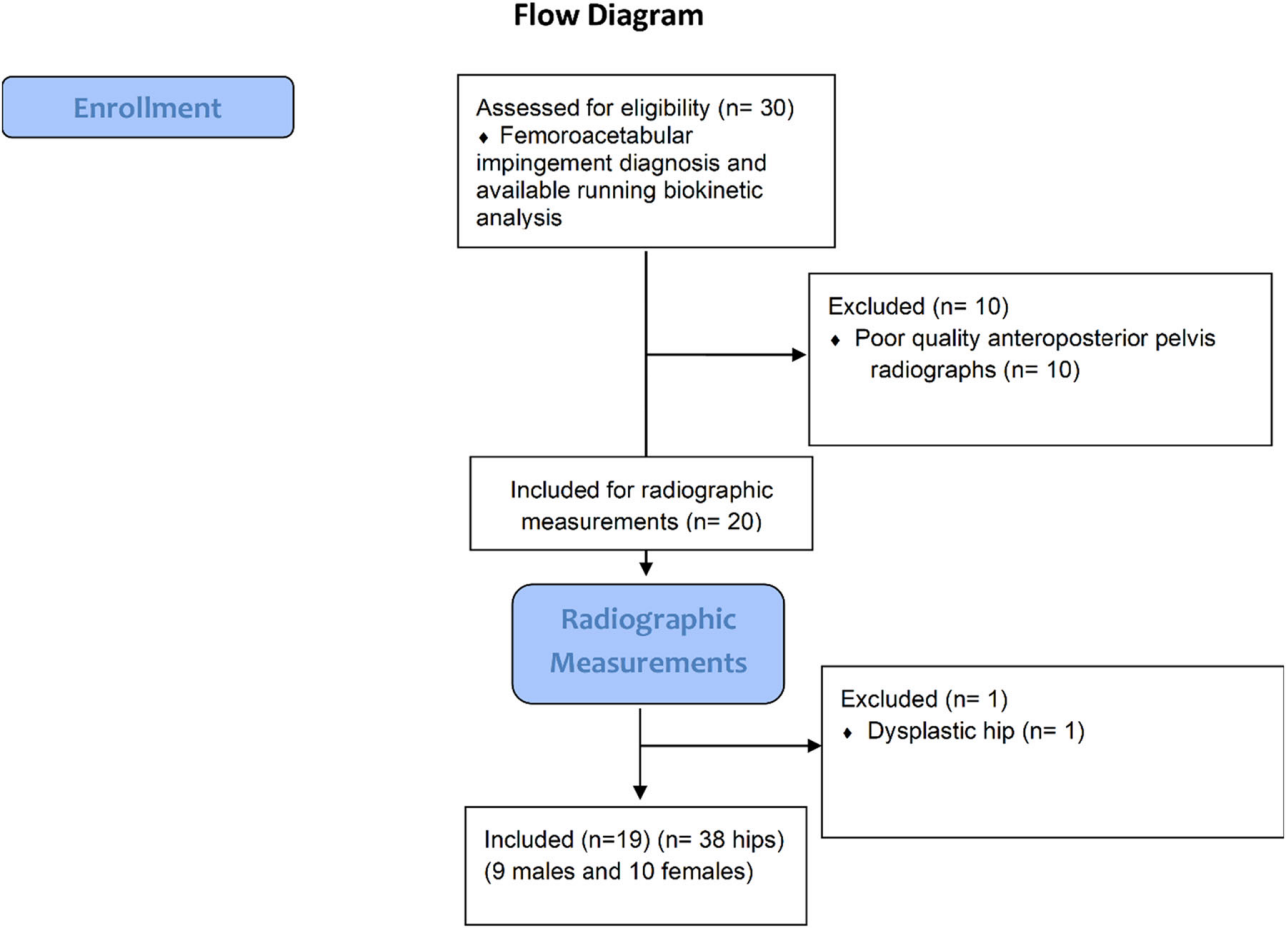
Flow diagram.

Of 30 patients with unilateral FAI diagnoses who underwent running biokinetic analysis, 20 patients (40 hips) had good‐quality AP pelvis radiographs and had consented to participate in this study (Figure 4). After the measurements on the standard radiographs, one patient was classified as dysplastic and then excluded. At the end, 19 patients (38 hips) remained in the study (9 males and 10 females); mean age was 40 ± 10 years, body weight: 73.3 ± 12.5 kg, height: 174.4 ± 9.0 cm and BMI: 24.0 ± 2.7 kg/m². The average PD and FAD used for image adjustment were 4.6° ± 3.8° and 5.3° ± 2.6°, respectively. Comparing symptomatic and asymptomatic sides, the presence of symptoms did not influence the PD magnitude (symptomatic: 4.5° ± 3.8°; asymptomatic: 4.8° ± 3.9°) in this group of patients (*t* = −0.194; *p* = 0.847). The mean FAD for the symptomatic limbs was 5.6° ± 2.2° and for the asymptomatic limbs was 5.1° ± 3.0°, without significant differences (*t* = 0.625; *p* = 0.536).

Comparing the adjusted with the standard radiographs, all measurements had a statistically significant variation (*p* < 0.05). The AI, SA, EI and FEAR index values increased (*p‐*value < 0.001 for all parameters), and the LCEA decreased (*p* < 0.001). The FEAR index had the highest variation during running (Table 1).

**Table 1:** Descriptive values (mean ± standard deviation) and comparison between standard and adjusted images.

	Standard	Adjusted	Variation	*t* or *W*	*p*‐value
LCEA (°)	31.7 ± 5.1	26.9 ± 6.6	−4.8 ± 4.1	7.16	<0.001*
AI (°)	3.8 ± 4.9	9.0 ± 6.3	5.2 ± 4.5	−7.17	<0.001*
SA (°)	41.3 ± 3.4	45.9 ± 5.3	4.7 ± 4.0	−7.19	<0.001*
EI (%)	14.5 ± 5.4	20.0 ± 7.0	5.4 ± 4.4	−7.60	<0.001*
FEAR index (°)	−17.6 ± 7.9	−7.5 ± 10.8	10.1 ± 5.9	−10.67	<0.001*

Abbreviations: AI, acetabular index; EI, extrusion index; FEAR index, femoro‐epiphyseal acetabular roof index; LCEA, lateral centre‐edge angle; SA, sharp angle.

* *p* < 0.05 for the comparison between standard versus manipulated.

When analysing the correlation between the variation of the radiographic measurements and the kinematic parameters, PD showed a very large correlation with the SA, AI, LCEA, EI and FEAR index (0.89, 0.88, −0.84, 0.79 and 0.73, respectively). FAD showed moderate correlation with the FEAR index and EI variation (0.47 and 0.31, respectively), and small correlation with LCEA (−0.22), respectively. These results are presented in Table 2.

**Table 2:** Correlation between the variation of the radiographic measurements (standard vs. adjusted) and the kinematic parameters.

	Pelvic drop	*p*‐value	Femoral adduction	*p*‐value
LCEA (°)	−0.84	<0.001*	−0.22	0.182
AI (°)	0.88	<0.001*	0.008	0.962
SA (°)	0.89	<0.001*	0.06	0.730
EI (%)	0.79	<0.001*	0.31	0.058
FEAR Index (°)	0.73	<0.001*	0.47	0.003*

Abbreviations: AI, acetabular index; EI, extrusion index; FEAR index, femoro‐epiphyseal acetabular roof index; LCEA, lateral centre‐edge angle; SA, sharp angle.

* *p* < 0.05 for the correlation tests.

**Figure 5:**
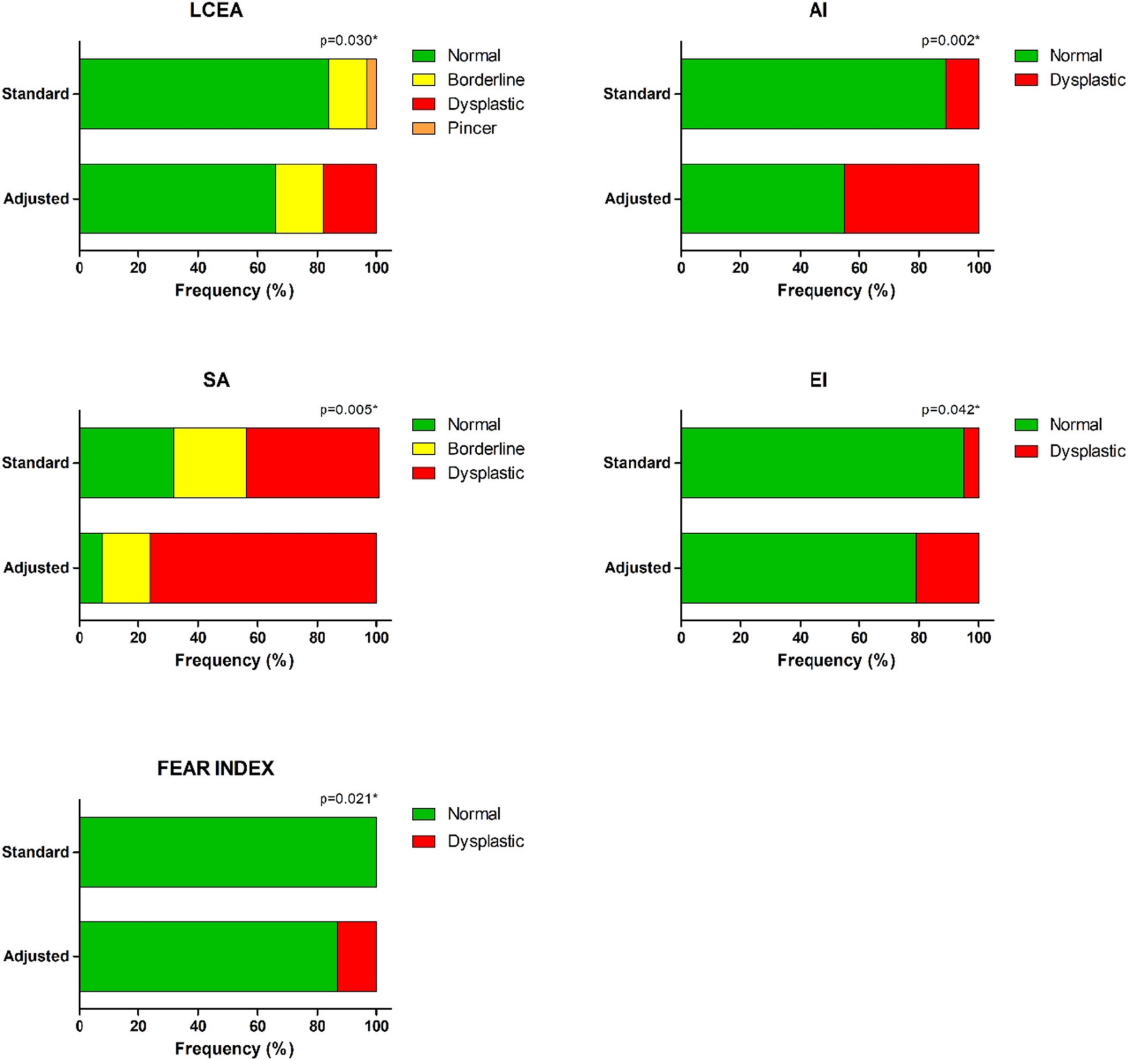
Proportion of dysplastic hips on the standard and adjusted images. AI, acetabular index; EI, extrusion index; FEAR index, femoro‐epiphyseal acetabular roof index; LCEA, lateral centre‐edge angle; SA, sharp angle; SD, standard deviation. *p < 0.05 for the chi‐squared test.

When comparing radiographic measurement classifications from standard to adjusted radiographs, 7 (18%) hips that were initially classified as having a normal LCEA value were reclassified as dysplastic after image adjustment. In the AI evaluation, the number of hips classified as dysplastic increased from 4 (11%) to 17 (45%) hips. Similarly, the SA classification demonstrated an increase in the number of hips considered dysplastic from 17 (45%) to 29 (76%) hips. Regarding the EI classification, the number of dysplastic hips also increased from 2 (5%) to 8 (21%) hips. Five (13%) became classified as unstable from standard to adjusted radiographs in the FEAR index classification (none were classified as unstable on standard radiographs) (Figure 5).

The linear regression models demonstrated that the PD was able to explain 70%, 77%, 78%, 61% and 52% (*p* < 0.001) of the variation in the LCEA, AI, SA, EI and FEAR index, respectively (Table 3). For the FEAR index, when adding the FAD in the regression model, the percentage of explanation in the variation increased to 65% (*p* < 0.001). In this multiple regression analysis, no issues related to multicollinearity were identified, as indicated by the weak correlation between PD and FAD (*r* = 0.14, *p* = 0.409), the low variance inflation factor (VIF = 1.02), and the high tolerance value (0.981). According to the regression model, each degree of PD was responsible for an increase of 1.13°, 1.04°, 0.94° and 0.92% of the FEAR index, AI, SA and EI, respectively. Moreover, the LCEA was found to decrease −0.92° for each degree of PD.

**Table 3:** Linear regressions (independent: pelvic drop, femoral adduction; dependent: variation).

	*R*	*R*²	Adjusted *R*²	*p*‐value	Equation	Interpretation
LCEA (°)	0.846	0.716	0.708	<0.001*	LCEA = −0.527 + (−0.923 * PD)	Each 1° increase in PD reduces LCEA by 0.9°.
AI (°)	0.882	0.778	0.772	<0.001*	AI = 0.398 + (1.044 * PD)	Each 1° increase in PD increases AI by 1.0°.
SA (°)	0.889	0.790	0.784	<0.001*	SA = 0.319 + (0.943 * PD)	Each 1° increase in PD increases SA by 0.9°.
EI (%)	0.793	0.628	0.618	<0.001*	EI = 1.181 + (0.921 * PD)	Each 1° increase in PD increases EI by 0.9°.
FEAR index (°)	0.732	0.535	0.523	<0.001*	FEAR index = 4.94 + (1.13 * PD)	Each 1° increase in PD increases the FEAR INDEX by 1.1°.
FEAR index (°)	0.819	0.671	0.652	<0.001*	FEAR index = 0.885 + (1.048 * PD) + (0.830 * FAD)	PD increases the FEAR INDEX by 1.0° per degree; FAD by 0.8° per degree.

Abbreviations: AI, acetabular index; EI, extrusion index; FAD, femoral adduction; FEAR index, femoro‐epiphyseal acetabular roof index; LCEA, lateral centre‐edge angle; PD, contralateral pelvic drop; SA, sharp angle.

* *p* < 0.001 for the regression analysis.

## DISCUSSION

The most important findings of the present study were that all evaluated radiographic parameters related to femoral head coverage in adjusted images demonstrated changes consistent with reduced coverage when compared to traditional radiographs. Further, this dynamic change resulted in functional dysplasia during running in some patients. When adjusted radiographic parameters were compared to static, the AI, SA, EI and FEAR index increased (*p‐*value of <0.001 for all parameters), and the LCEA decreased (*p* < 0.001). Regarding dysplasia classification, an increased number of hips were classified as dysplastic in the adjusted radiographs compared to standard images based on all radiographic measures (LCEA, AI, EI, SA evaluation and FEAR index classification). Moreover, these results suggest that dynamic pelvic mechanics, particularly the contralateral PD during running, are strongly associated with reductions in femoral head coverage. The regression model revealed that each degree of PD can decrease the LCEA by −0.92° and increase the FEAR index, AI, SA and EI by 1.13°, 1.04°, 0.94° and 0.92%, respectively. All these findings suggest a possible influence of the peak of PD and FAD on the radiographic femoral head bone coverage parameters, generating hip instability that was not detected on static images.

Historically, the quantification of femoral head coverage has been based on the LCEA since the description in 1939 [41]. Even in the absence of symptoms, the magnitude of the LCEA has been linked to the progression of OA [27]. Thomas et al showed a 13% increased likelihood of developing OA for each 1° loss of lateral coverage below 28° [38]. The cut‐off point to consider a hip as dysplastic has been subject to extensive discussion without consensus. The current data showed an average reduction of 4.8 ± 4.1 (*p* < 0.001) in the PD during running, with a very large correlation (−0.84) between PD and reduction of LCEA. Seven (18%) hips with previous values above 20° became considered dysplastic after image adjustment for PD. Additionally, the increase of 5.4 ± 4.4 in the EI also denotes a decrease in the femoral head bone coverage during the peak of PD. Moreover, running at 3.5 m/s produces a joint contact force of 5.2 + /−0.4 of body weight during the stance phase [2]. Coincidentally, that is also when the peak of PD occurs. Therefore, the moment of highest load on the femoral head during running is also the moment of lowest femoral head bone coverage. This increases contact pressure over a reduced area of articular cartilage. The reclassification of hips as dysplastic in the present study may have important clinical implications for active individuals like runners, who are exposed to higher vertical joint loads compared to sedentary patients. These findings could influence diagnostic accuracy and treatment decisions in this population.

Excessive PD and FAD in runners are biomechanical alterations closely linked to different types of orthopaedic injuries with clinical significance. Bramah et al reported orthopaedic injured patients having contralateral PD and FAD of 6.4° ± 2.1° and 13.0° ± 3.9° (*p* < 0.01), respectively. On the other hand, healthy control runners showed a PD of 3.7° ± 1.9° and a FAD of 6.4° ± 2.1° [4]. The current study, including only unilateral FAIS patients, the PD and FAD were 4.6° ± 3.8° e 5.3° ± 2.6°, respectively. Contrasting with the previous study, the presence of symptoms did not influence the PD magnitude in this group of patients (*t*_(36)_ = −0.194; *p* = 0.847). However, it is important to note that we also analysed the asymptomatic contralateral side, which may limit the comparability with studies in the literature that assess only FAI hips.

A considerable number of studies have been published in the last few decades focusing specifically on borderline dysplasia definition, surgical indications and outcomes [12, 17, 19, 20, 32, 40]. Treatment strategies have been challenged by the incomplete characterisation of the pathology of borderline hip dysplasia. When considering all radiographic measures of acetabular coverage, patients with LCEA 18°–25° may still demonstrate high variability in other radiographic measures of femoral head coverage, which may result in unpredictable outcomes, especially with hip arthroscopy [40]. However, a recent systematic review demonstrated comparable short‐ to mid‐term outcomes between PAO and hip arthroscopy, underscoring the need for standardised borderline dysplasia definitions [30]. The current study methodology brings additional factors to consider in the evaluation of a patient with borderline dysplasia. The peak contralateral PD during running demonstrated a very large correlation with the variation in all five measurements evaluated, resulting in a reduction of femoral head bone coverage. The linear regression models confirmed this relationship, showing that each degree of PD can be responsible for a decrease of −0.92° in the LCEA and for an increase of 1.13°, 1.04°, 0.94° and 0.92% of the FEAR index, AI, SA and EI, respectively. This demonstrated a possible occurrence of functional dysplasia in a substantial percentage of hips, caused by excessive pelvic motion in the coronal plane, which is not captured on standard radiographs. Moreover, the dynamic radiographic measurements reproducing the impact of the PD and FAD might be paramount in the decision of whether borderline dysplastic hips can be successfully treated arthroscopically.

Regarding hip stability, the FEAR index, which comprises the relationship between the central one‐third of the physeal scar and the sourcil and may serve as a developmental marker. The first study on this subject presented that hips with a LCEA of 25° or less and a FEAR index less than 5° are likely to be stable [42]. Subsequently, another cohort stated that a FEAR index greater than −1.3° was associated with hip dysplasia and a FEAR index less than −1.3° was associated with FAIS [35]. This study used the original description of 5° as a reference and showed 5 (13%) hips becoming classified as unstable during running. The FEAR Index had the highest variation between measurements (10.1° ± 5.9°, *p* < 0.001). This finding can be explained by the movement of the central one‐third of the physeal scar and the sourcil in opposite directions at the peak of contralateral PD and ipsilateral FAD during the stance phase of running. This also confirms that motion may predispose to instability, particularly in the coronal plane.

Importantly, the term functional dysplasia is used in this study descriptively and does not represent a new diagnostic classification. Rather, it refers to a kinematically driven reduction in apparent femoral head coverage, whereby traditional AP radiographic parameters shift into ranges associated with dysplasia when pelvic and femoral kinematics observed during running, particularly contralateral PD and associated FAD, are applied. This finding may represent a normal, transient phenomenon in some individuals, while in others, especially symptomatic patients with borderline dysplasia, it may be clinically relevant. At present, the relationship between these findings and clinical outcomes remains unknown and warrants further investigation.

The present study evaluated the influence of peak PD on 2D parameters of femoral head osseous coverage, using commonly employed radiographic measurements rather than 3D assessments. Although three‐dimensional CT is unquestionably superior to two‐dimensional measurements for defining femoral head bone coverage [18, 31], understanding how motion influences radiographic parameters will provide the foundation for future studies with more detailed assessments. Previous studies have evaluated femoral head bone coverage during gait using 3D methods [3, 39]. Uemura et al. assessed asymptomatic individuals using dual fluoroscopy combined with three‐dimensional models derived from computed tomography scans to compare acetabular bone coverage in the supine position and during gait [39]. Their findings demonstrated minimal variation in total femoral head coverage throughout the gait cycle. Another study compared 3D femoral head bone coverage in patients with developmental dysplasia of the hip before and after periacetabular osteotomy (PAO) [3]. Post‐PAO, coverage in the posterolateral region was greater during 37%–48% of the gait cycle (*p* ≤ 0.012). However, the motion simulations in that study were based on mean values obtained from a previous investigation. Although the results were not individualised to the subjects included in the study, the work from Bourantas et al. provides early evidence of how a commonly used surgical procedure for dysplasia (PAO) may influence dynamic femoral head coverage during gait [3].

This study has several limitations. First, our methodology was unable to reproduce hip coronal translation during motion on the radiographic manipulations. In the literature search, no reference regarding femoral translation during running was found; however, a previous study on asymptomatic patients reported that hip translation during gait was around 0.6 mm. Taking femoral head translation into consideration may influence the results of this study [10]. Second, no movement variation on the axial and sagittal plane was considered; only the PD and FAD peak angles on the coronal plane were used as the reference for radiograph adjustment. Third, the study exclusively utilised coronal plane data from the 3D running biokinetic analysis as the reference for radiograph manipulation. Future studies considering motion in all three planes are necessary. Fourth, studies involving kinematic measurements may be subject to alterations due to errors in marker placement on the skin. Fifth, only recreational runners with unilateral symptomatic FAIS were included. Sixth, no muscle strength, training status and axial alignment data were available, and these factors may influence the PD and FAD. Lastly, due to the limited sample size and the specific characteristics of the sample, the results cannot be fully transferred to the general population with FAIS.

## CONCLUSION

This study demonstrated that the femoral head acetabular coverage significantly decreased due to peak contralateral PD during running. This resulted in an increase in dysplastic findings on adjusted radiographs. Patients with significant contralateral PD and ipsilateral FAD during running may demonstrate kinematically driven reductions in femoral head coverage, consistent with the concept of functional dysplasia. Future studies should investigate the clinical consequences of functional dysplasia.

## AUTHOR CONTRIBUTIONS

Renato Locks contributed to conceptualisation, data collection, supervision, writing of the original draft and manuscript revision. Eliane C. Guadagnin handled data curation, methodology and manuscript review. Felipe F. Gonzalez was involved in data collection, formal analysis, writing of the original draft and manuscript review. Guilherme Pradi Adam was involved in the conceptualisation, data collection and manuscript review. Jorge Chahla contributed to the investigation and manuscript review. Andrew E. Jimenez was responsible for manuscript review and data visualisation. Robert F. LaPrade provided supervision and manuscript review. Leonardo Metsavaht contributed to conceptualisation, provided supervision and manuscript review. Gustavo Leporace contributed to conceptualisation, data analysis, supervision and manuscript review.

## CONFLICT OF INTEREST STATEMENT

Renato Locks: The author has no relevant financial or nonfinancial interests to disclose. Eliane C. Guadagnin: The author has no relevant financial or nonfinancial interests to disclose. Felipe F. Gonzalez: The author has no relevant financial or nonfinancial interests to disclose. Guilherme Pradi Adam: The author has no relevant financial or nonfinancial interests to disclose. Jorge Chahla: Board membership of the American Orthopaedic Society for Sports Medicine, Arthroscopy Association of North America and International Society of Arthroscopy Knee Surgery and Orthopaedic Sports Medicine. Consulting or advisory of Arthrex Inc; CONMED Corp, Ossur Americas and Smith and Nephew Inc. Speaking and lecture fees of Smith and Nephew Inc. Andrew E. Jimenez: Board membership of American Journal of Sports Medicine (AJSM). Consulting or advisory of Smith and Nephew Inc. Research committee: International Society of Hip Arthroplasty (ISHA). Robert F. LaPrade: Consultant of Smith and Nephew and Ossur. Royalties: Smith and Nephew; Ossur; and Elsevier. Research grants: AANA; AOSSM; Smith and Nephew; and Ossur. Leonardo Metsavaht: The author has no relevant financial or nonfinancial interests to disclose. Gustavo Leporace: The author has no relevant financial or nonfinancial interests to disclose.

## ETHICS STATEMENT

Escola Paulista de Medicina, Universidade Federal de São Paulo. Project N°.: 6.951.290.

## Supporting information

STROBE‐checklist‐v4‐combined‐PlosMedicine_2025_09_09.

## Data Availability

The data that support the findings of this study are available from the corresponding author upon reasonable request.
