# Supplementary material for: Introducing functional dysplasia: Dynamic pelvic mechanics during running reduce femoral head coverage
Source: J Exp Orthop. 2026 Apr 14;13(2):e70711. doi: 10.1002/jeo2.70711 (PMC13078133; doi:10.1002/jeo2.70711)
Supplement: Supplementary file 1 — STROBE‐checklist‐v4‐combined‐PlosMedicine_2025_09_09. [file JEO2-13-e70711-s001.docx]

STROBE Statement—checklist of items that should be included in reports of ***cross-sectional studies***

|  | **Item No.** | **Recommendation** | **Page No.** | **Relevant text from manuscript** |
| --- | --- | --- | --- | --- |
| **Title and abstract** | 1 | (*a*) Indicate the study’s design with a commonly used term in the title or the abstract | 2 | Abstract: "Study design: Cross-Sectional Study" |
|  |  | (*b*) Provide in the abstract an informative and balanced summary of what was done and what was found | 2 | The abstract includes background, purpose, study design, methods, results, and conclusion. For example: "Participants underwent three-dimensional (3D) running analysis and AP pelvis radiographs... Patients with significant PD and FAD during running may be at risk for functional dysplasia." |
| **Introduction** |  |  |  |  |
| Background/rationale | 2 | Explain the scientific background and rationale for the investigation being reported | 4 | "The literature reports that FAIS and acetabular dysplasia are the two main causes of early hip degeneration (Leunig & Ganz, 2014)." |
| Objectives | 3 | State specific objectives, including any prespecified hypotheses | 5 | "The purpose of this study was to investigate the influence of peak contralateral PD and FAD during running..." |
| **Methods** |  |  |  |  |
| Study design | 4 | Present key elements of study design early in the paper | 2 | "This cross-sectional retrospective study..." |
| Setting | 5 | Describe the setting, locations, and relevant dates, including periods of recruitment,  exposure, follow-up, and data collection | 5 | “...patients who had undergone biokinetic analysis and standardized supine AP pelvic radiographs… between 2020 and 2023… in our institution.” |
| Participants | 6 | (*a*) *Cohort study*—Give the eligibility criteria, and the sources and methods of selection of participants. Describe methods of follow-up  *Case-control study*—Give the eligibility criteria, and the sources and methods of case ascertainment and control selection. Give the rationale for the choice of cases and controls *Cross-sectional study*—Give the eligibility criteria, and the sources and methods of selection of participants | 5 | "Patients who had undergone biokinetic analysis and standardized supine AP pelvic radiographs, with a prior diagnosis of symptomatic unilateral FAIS on the records of our institution between 2020 and 2023, were invited to provide informed consent to participate in this study... Patients with previous hip or spine surgeries, signs of hip OA (Tonnis Grade >1) and dysplasia (LCEA<20o), and previously documented lower limb length discrepancies greater than 1 cm were excluded to avoid any factor that could influence the pelvic and lower limb biomechanics. Also, in the same way, patients with poor-quality radiographs according to the European guidelines on quality criteria for diagnostic radiographic images (European Commission, 1996)." |
|  |  | (*b*) *Cohort study*—For matched studies, give matching criteria and number of exposed and unexposed  *Case-control study*—For matched studies, give matching criteria and the number of controls per case |  |  |
| Variables | 7 | Clearly define all outcomes, exposures, predictors, potential confounders, and effect modifiers. Give diagnostic criteria, if applicable | 5, 6, 7, 8, 9, 10 | “The variation index for all 5 measurements was obtained by subtracting the standard by the adjusted radiographic values…” “The LCEA, AI, SA, EI, and FEAR index were calculated and classified…” “...PD and FAD during running…” |
| Data sources/ measurement | 8* | For each variable of interest, give sources of data and details of methods of assessment (measurement). Describe comparability of assessment methods if there is more than one group | 5, 6, 7, 8, 9, 10 | Detailed descriptions of the biomechanical data collection protocol with Qualysis cameras, acquisition and adjustment of radiographs using Photoshop, and measurements performed by an experienced radiologist using Carestream software. |
| Bias | 9 | Describe any efforts to address potential sources of bias | 5, 6, 7, 8, 9, 10 | “The images were numbered and shared with the investigators without any patient identification…” “All measurements were performed twice, blinded to each other...” |
| Study size | 10 | Explain how the study size was arrived at | 10 | “The sample size was determined using the data… from a previously published study… to determine the effect size... nine hips were the minimal number... to achieve 80% statistical power with an alpha level of 0.05.” |

Continued on next page

| Quantitative variables | 11 | Explain how quantitative variables were handled in the analyses. If applicable, describe which  groupings were chosen and why | 8, 9, 10 | “Means, standard deviations and frequencies were utilized…” “To compare standard versus adjusted images, paired t-tests or Wilcoxon tests were performed…” |
| --- | --- | --- | --- | --- |

Statistical 12 (*a*) Describe all statistical methods, including those used to control for confounding methods (*b*) Describe any methods used to examine subgroups and interactions

1. Explain how missing data were addressed

8, 9, 10

1. *Cohort study*—If applicable, explain how loss to follow-up was addressed

“Shapiro-Wilk test for normality… Pearson correlation... chi-squared test… linear regressions… significance level of 5%... analyses performed with Jamovi software.”

*Case-control study*—If applicable, explain how matching of cases and controls was addressed *Cross-sectional study*—If applicable, describe analytical methods taking account of sampling strategy

|  |  | (*e*) Describe any sensitivity analyses |
| --- | --- | --- |
| **Results** |  |  |

Participants 13* (a) Report numbers of individuals at each stage of study—eg numbers potentially eligible, examined for eligibility, confirmed eligible, included in the study, completing follow-up, and analysed

Of 30 patients with unilateral FAI who underwent running biokinetic analysis, 20 patients (40 hips) had adequate radiographs and agreed to participate. After excluding one dysplastic patient, 19 patients (38 hips) remained in the study.

11

?

CONSORT flow diagram

11

10 patients were excluded due to inadequate radiographs; 1 excluded after classification as dysplastic.

(

c)

Consider use of a flow diagram

(b) Give reasons for non-participation at each stage

Descriptive data 14*

Mean age: 40 ± 10 years; body mass: 73.3 ± 12.5 kg; height: 174.4 ± 9.0 cm; BMI: 24.0 ± 2.7 kg/m². Mean pelvic drop (PD): 4.6 ± 3.8°; femoral adduction (FAD): 5.3 ± 2.6°.

(a) Give characteristics of study participants (eg demographic, clinical, social) and information on exposures and potential confounders

11

1. Indicate number of participants with missing data for each variable of interest
2. *Cohort study*—Summarise follow-up time (eg, average and total amount)

Outcome data 15* *Cohort study*—Report numbers of outcome events or summary measures over time

All radiographic measurements (AI, SA, EI, FEAR index, LCEA) showed significant changes after adjustment. Example: dysplastic LCEA increased from 0 to 7 hips, AI from 4 to 17, SA from 17 to 29, EI from 2 to 8, and FEAR from 0 to 5. AI: acetabular index; SA: sharp angle; EI: extrusion index; FEAR index: femoro-epiphyseal acetabular roof index; LCEA: lateral center-edge angle.

12

*Case-control study—*Report numbers in each exposure category, or summary measures of exposure

*Cross-sectional study—*Report numbers of outcome events or summary measures

Main results 16 (*a*) Give unadjusted estimates and, if applicable, confounder-adjusted estimates and their precision

13, 14

"The linear regressions showed that the PD was able to explain 71%, 77%, 78%, 62% and 52% (p<0.001) of the variation in the LCEA, AI, SA, EI and FEAR INDEX, respectively..." "...when adding the FAD in the regression together with the PD, the percentage of explanation in the variation increased to 65% (p<0.001)."

(eg, 95% confidence interval). Make clear which confounders were adjusted for and why they were included

(*b*) Report category boundaries when continuous variables were categorized

|  |  | (*c*) If relevant, consider translating estimates of relative risk into absolute risk for a meaningful time  period |
| --- | --- | --- |

Continued on next page

"...PD showed a very large correlation with the SA, AI, LCEA, EI and FEAR index (0.89, 0.88, -0.84, 0,79 and 0,73, respectively)."

11, 13

| Other analyses 17 | Report other analyses done—eg analyses of subgroups and interactions, and sensitivity analyses |
| --- | --- |
| **Discussion** | "...all radiographic parameters related to femoral head coverage demonstrated changes consistent with reduced coverage..." "...results suggest that dynamic pelvic mechanics, particularly the contralateral PD during running, are strongly associated with reductions in femoral head coverage." |
| Key results 18 | Summarise key results with reference to study objectives |
| Limitations 19 | Discuss limitations of the study, taking into account sources of potential bias or imprecision. Discuss  Limitations included lack of hip translation modeling, no axial/sagittal plane consideration, reliance on skin markers, and a limited sample of recreational runners with unilateral FAIS.  14  both direction and magnitude of any potential bias |
| Interpretation 20 | Give a cautious overall interpretation of results considering objectives, limitations, multiplicity of  The discussion compared current findings with historical data and recent studies, including those on borderline dysplasia and FEAR index, cautiously suggesting the clinical relevance of “functional dysplasia” in active populations.  16, 17  analyses, results from similar studies, and other relevant evidence |
| Generalisability 21 | Discuss the generalisability (external validity) of the study results  The authors state that due to the specific sample (recreational runners with unilateral FAIS), the findings may not be generalisable to the broader FAIS population  16 |
| **Other information** | 16 |
| Funding 22 | Give the source of funding and the role of the funders for the present study and, if applicable, for the  original study on which the present article is based |

NR

--

*Give information separately for cases and controls in case-control studies and, if applicable, for exposed and unexposed groups in cohort and cross-sectional studies.

**Note:** An Explanation and Elaboration article discusses each checklist item and gives methodological background and published examples of transparent reporting. The STROBE checklist is best used in conjunction with this article (freely available on the Web sites of PLoS Medicine at http://www.plosmedicine.org/, Annals of Internal Medicine at http://www.annals.org/, and Epidemiology at http://www.epidem.com/). Information on the STROBE Initiative is available at www.strobe-statement.org.
